# Deafness alters the spatial mapping of touch

**DOI:** 10.1371/journal.pone.0192993

**Published:** 2018-03-02

**Authors:** Andréanne Sharp, Simon P. Landry, Maxime Maheu, François Champoux

**Affiliations:** École d’orthophonie et d’audiologie, Université de Montréal, Montréal, Québec, Canada; Universidad de Salamanca, SPAIN

## Abstract

Auditory input plays an important role in the development of body-related processes. The absence of auditory input in deafness is understood to have a significant, and even irreversible, impact on these processes. The ability to map touch on the body is an important element of body-related processing. In this research, the crossed-arm temporal order judgment (TOJ) task was used to evaluate the spatial mapping of touch. This task elicits a conflict between visual and somatosensory body-related information through a change in posture. We used the crossed-arm TOJ task to evaluate the spatial mapping of touch in deaf participants. Results suggested that a change in posture had a greater impact on congenitally deaf participant TOJ than for hearing participants. This provides the first evidence for the role of early auditory exposure on spatial mapping of touch. More importantly, most deaf participants had auditory prosthetics which provided auditory input. This suggests an important, and possibly irreversible, impact of early auditory deprivation on this body-related process.

## Introduction

The representation and control of our body relies on the ability to perceive and distinguish our limbs in space, independent of their posture [1]. This process requires the interaction of somatosensory and retinotopic inputs [2]. Somatosensory inputs provide information on the body’s position in relationship to itself. This sensory information forms the internal frame of reference. Retinotopic input provides information on the body’s position in relationship to the environment. This sensory information forms the external frame of reference. Combining these complementary frames of reference forms a body representation that allows us to interface with our surroundings [2].

The crossed-arm TOJ task [3], [4] is a complex tactile task used to study the spatial mapping of touch by creating a conflict between internal and external frames of reference. In it, participants are asked to identify the laterality of the hands to be first stimulated (left hand or right hand) using buttons placed under their feet. Participants must perform this with their arms either uncrossed or crossed. For the crossed-arm condition stimulating the right hand first requires the participant to respond with the left foot, as the hand is located left of the body-midline. This creates a conflict between the information from internal somatosensory and external retinotopic frames of reference. Perceptual results from the task are compared using the cumulated percentage of correct responses over all stimulus onset asynchronies (SOA) in uncrossed and crossed conditions. This can then be analyzed to determine the presence of a significant increase of the TOJ error rate when crossing the arms [5].

The crossed-arm TOJ task is a multisensory task where vision plays an important role along with touch (see, e.g., [6–8]). Vision provides such crucial information for this task that the crossed-arm deficit can be nearly eliminated by simply seeing uncrossed rubber hands [9]. Results from previous investigations suggest that the interaction between frames of reference develops in early infancy through sensory and motor experience [10, 11]. Moreover, congenitally blind participants were found to have significantly lower TOJ error rates when crossing the arms [8]. Studies have since tested the crossed-arm TOJ task with blindfolded participants [12–14]. Results from these short-term sensory deprivation studies have revealed that temporary visual deprivation does not seem to have a similar effect on the task as congenital blindness since TOJ errors were not significantly different from the control group. This suggests that early sensory exposure plays an essential role in the development of the automatic interaction of internal and external coordinates for touch processing.

A recent investigation by Noel & Wallace [15] on the impacts of temporary sensory deprivation revealed a significant increase to crossed-arm TOJ error rate for auditory deprivation. In their study, participants were temporarily deprived of audition, vision, or both and performed the TOJ task. Their results suggest that auditory and audiovisual deprivation led to a significant increase in error rates, while visual deprivation did not lead to significant changes. It would seem that audition plays a crucial, but under studied, role in the spatial mapping of touch. Indeed, there are several evidences of disturbed neural representation of the body following deafness (for a review, see ref [16]). In this study, we investigated spatial mapping of touch in the deaf using crossed-arm TOJ task [17, 18]. We calculated the results from the crossed-arm TOJ task using the proportion correct difference (PCD). The PCD is a reliable performance metric that provides information on uncrossed and crossed responses error rates in a single score [17]. A low PCD represent a similar error rate between the crossed and uncrossed posture, while a higher PCD represents a large difference in error rates between postures. Due to the demonstrated importance of auditory input on the spatial mapping of touch [15], we hypothesise that crossing the arms will lead to a significantly greater error rate in deaf participants. This increase in error rates will be reflected by a higher means group PCD score.

## Materials and methods

### Participants

13 deaf (9 women, 4 men, mage = 38.4 years, range: 29–57 years) and 13 hearing group participants (9 women, 4 men, mage = 33.4, range = 20–59 years) took part in the study (see Table 1 for more details). Participants underwent a hearing test and a comprehensive vestibular evaluation by a certified audiologist. Two deaf participants chose to opt out of the vestibular evaluation. Hearing thresholds were determined using an audiometer (Astera, GN Otometrics, Denmark). All deaf participants suffered from congenital profound bilateral hearing loss (mean hearing thresholds from 250 Hz to 8 kHz > 100 dBHL). Hearing group pure-tone detection thresholds at octave frequencies ranging from 250 to 8000 kHz were within normal limits in both ears (mean hearing thresholds from 250Hz to 8kHz: 4.44±0.91 dBHL). A comprehensive peripheral vestibular evaluation of all six semi-circular canals using the video head impulse test (vHIT: Eyeseecam, Interacoustics, Denmark), both saccules with the cervical vestibular evoked myogenic potential (cVEMP: Eclipse EP-25/VEMP Interacoustics, Denmark) and both utricules using ocular vestibular evoked myogenic potential (oVEMP: Eclipse EP-25/VEMP Interacoustics, Denmark) was performed. We identified 7 deaf participants with a vestibular deficit of the 11 tested (64%). This proportion is consistent with previous studies assessing the vestibular function of hearing impaired participants, with or without cochlear implants (see, e.g., [19]–[23]). All other participants (including normal-hearing participants) had normal bilateral vestibular function. Twelve deaf participants communicated primarily through oral language and lip reading, and used auditory amplification (mean age of acquisition of hearing aids: 5.9 years ± 5.2). One deaf participant communicated primarily through sign language. Twelve deaf participants used hearing aids (mean age of acquisition: 5.9 years ± 5.2) and four used cochlear implants (mean age of acquisition: 40.3 years ± 9,7). The Research Committee for Health Sciences of the University of Montreal and the Center for Interdisciplinary Research in Rehabilitation of Greater Montreal approved all procedures and each participant provided written informed consent. All experiments were performed in accordance with relevant guidelines and regulations.

**Table 1:** Deaf participant’s individual characteristics.

Participant	Age	Sex	Duration of deafness (years)	Duration of hearing aid use (years)	Duration of cochlear implant use (years)
Deaf 1	29	M	29	26	1
Deaf 2	52	M	52	37	3
Deaf 3	51	F	51	48	4
Deaf 4	41	F	30	29	4
Deaf 5	32	F	32	26	N/A
Deaf 6	37	F	36	36	N/A
Deaf 7	34	F	34	31	N/A
Deaf 8	34	F	34	20	N/A
Deaf 9	33	F	33	29	N/A
Deaf 10	34	M	34	31	N/A
Deaf 11	29	F	29	28	N/A
Deaf 12	57	F	57	25	N/A
Deaf 13	36	M	30	20	N/A

### Procedure and stimuli

As previously used in our laboratory [24], we used a crossed-arm TOJ task [17, 18] and recorded non-speeded reaction times [3], [25, 26]. Participants held 4cm3 foam cubes between both thumbs and indexes. For each trial, 20-ms vibrations were delivered to each foam cube. Stimulations were separated by a variable stimulus onset asynchrony (SOA): ± 400, ± 200, ± 100, ± 50 ms, where negative SOAs indicated that the vibration was presented to the left index first. Two 20 trial practice blocks, one in each posture, were performed before the start of the experiment. Each of the eight SOAs were presented randomly 40 times over 20 blocks. Arm posture alternated between crossed and uncrossed for each block. Starting postures were counterbalanced across participants; odd numbered participants started in an uncrossed posture, even numbered participants started in a crossed posture. Two response buttons, one under each foot, were used to record participant responses. Participants were instructed to indicate the side of the cube having first vibrated with the response buttons, regardless of the arm posture.

Participant comprehension was verbally confirmed many times throughout the procedure. The task was first explained before the practice blocks. Participants were instructed to press on the pedal located on the same side of space as the cube that first vibrated. It was further specified that the side in space of the cube and the hand would be different for the crossed posture and that the side in space was the correct answer. Participants then performed the practice blocks with the experimenter at their side, clarifying any ambiguities and confirming their understanding of the task. Furthermore, task comprehension was verbally verified for all participants after the first crossed and uncrossed postures. Participants demonstrating a misunderstanding of the task during this last comprehension evaluation were eliminated from analysis. All participants confirmed their correct understanding of the task demand.

### Analysis

A PCD score was calculated to compare the crossed and uncrossed error rates [17, 18, 27]. The PCD is a single performance metric that reliable represents the entire curve of uncrossed and crossed responses [17]. This value is calculated by summing up the differences between the proportion of correct response for the crossed and uncrossed postures at each SOA. PCD scores range from 0 to 8. A score of 0 represents the exact same response for uncrossed and crossed postures at each SOA. This would occur if the participant were completely accurate in the uncrossed and crossed postures. A score of 8 represents exact opposite response for uncrossed and crossed postures at each SOA. This would occur if the participant were completely accurate in the uncrossed posture and completely inaccurate in the crossed posture. An indepedant t-test between mean group PCD will be used as statistical test. Furthermore, a repeated measure analysis will be used to compare unspeeded reaction times with two within subject factors (SOA and posture) and the between subject factor group. All analysis will be done with IBM SPSS statistics 23 software.

## Results

**Fig 1:**
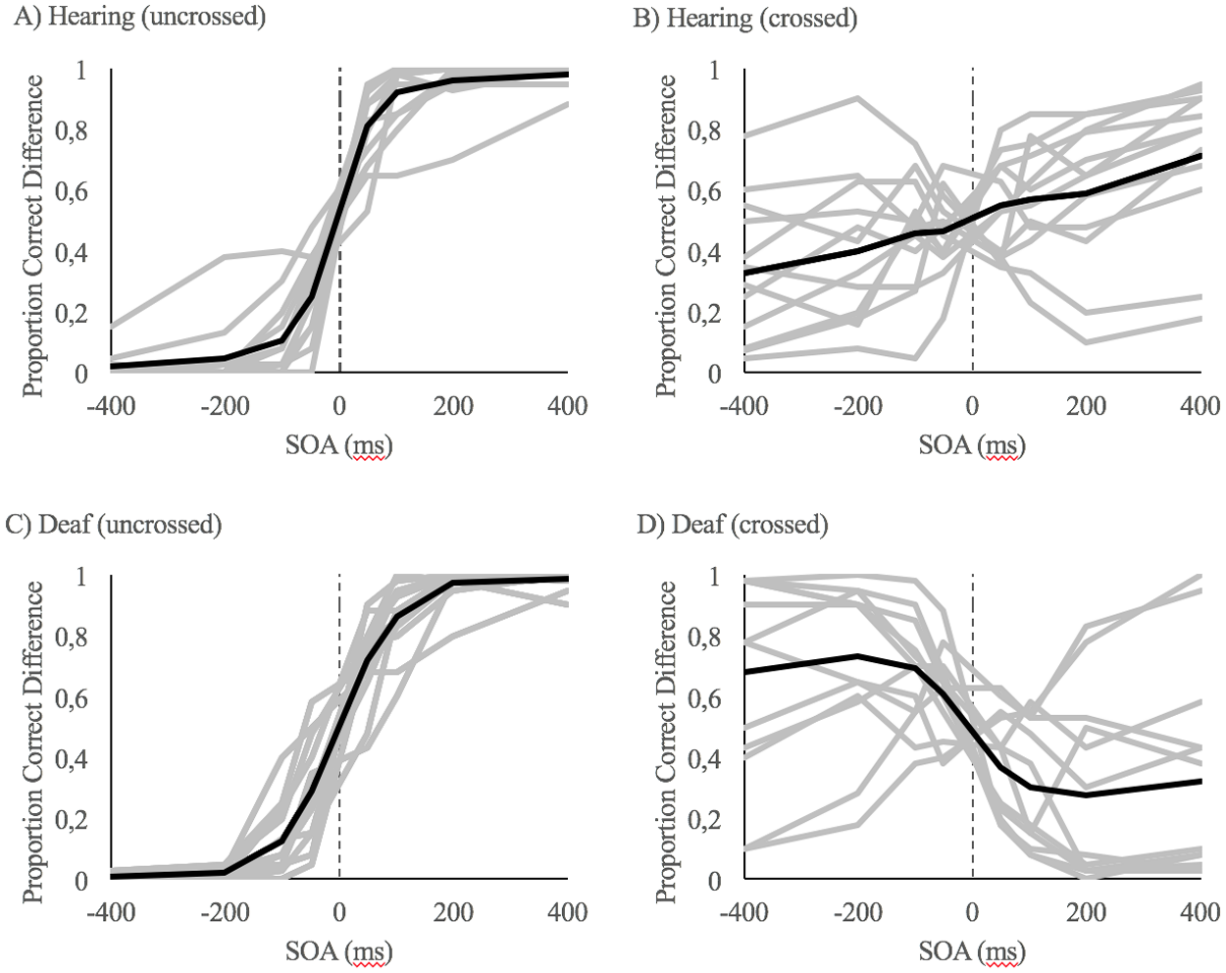
Proportion of right-hand first answers across different SOA. —SOA represent left presented first and + SOA represent right presented first. Individual scores are the gray lines and the group mean is the black line. A) Hearing control group in the uncrossed posture. B) Hearing control group in the crossed-arm posture. C) Deaf group in the uncrossed posture. D) Deaf group in the crossed-arm posture.

**Fig 2:**
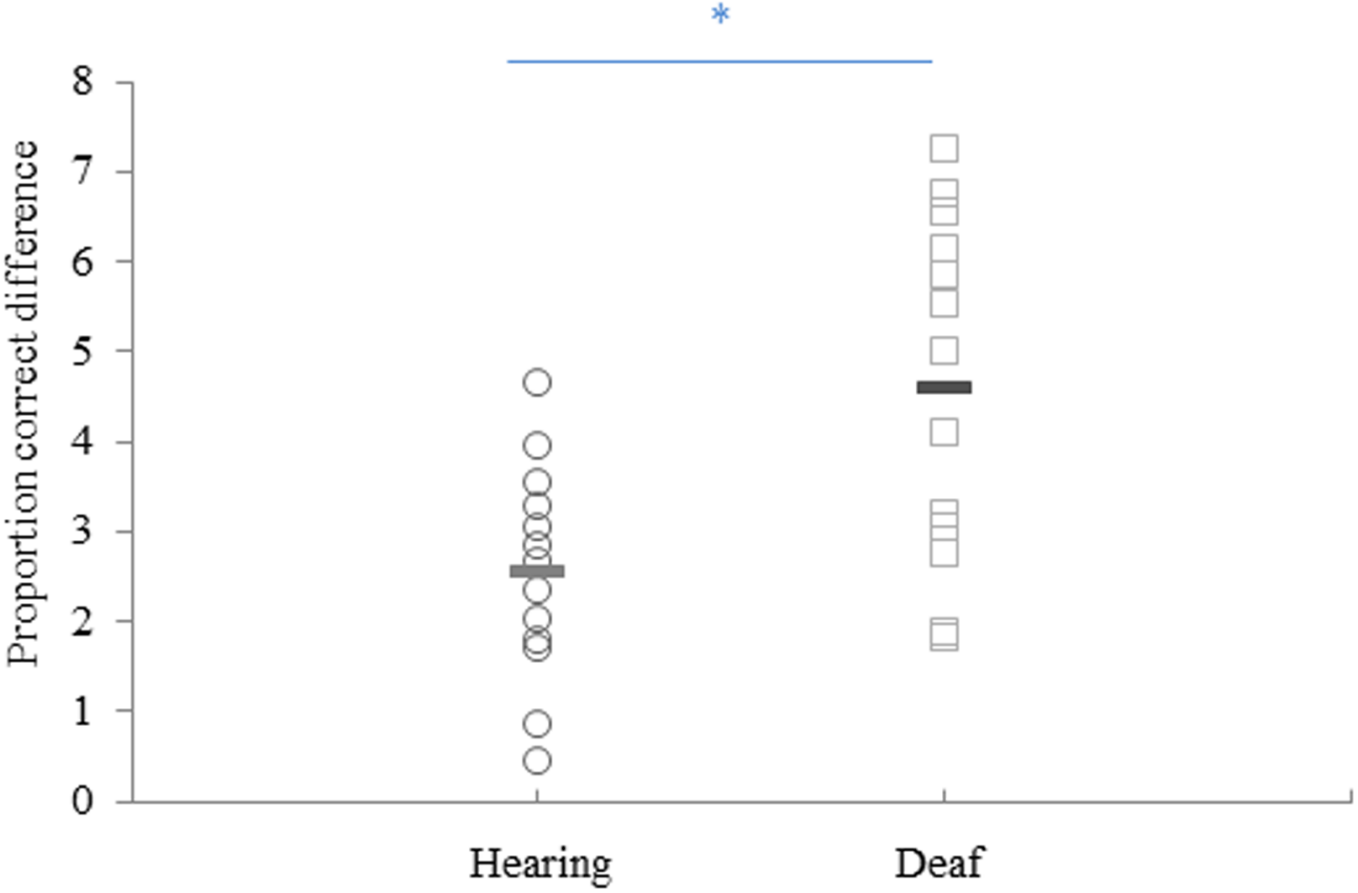
Individual PCD scores for hearing (n = 13), deaf participants (n = 13). Circles represent individual PCD scores for hearing and squares individual PCD scores for deaf. Lines represent mean scores calculated for each group. * represents *p* = .003.

Individual scores for proportion of right-hand first answers across different SOA are shown in Fig 1. We performed an independent t-test between the mean group PCD score for deaf (*M* = 4.59, *SD* = 1.91) and hearing group participants (*M* = 2.49, *SD* = 1.19). Results from this analysis suggested significant different PCD scores between groups (*t*(20.146) = -3.373, *p* = .003). This result indicates that deaf participants had significantly higher TOJ error rates for the crossed posture (see Fig 2). Mean group unspeeded reaction times were analyzed using a repeated measure analysis with two within subject factors (SOA and posture) and the between subject factor group. Results from this analysis failed to reveal a significant between-subjects effect of group for reaction times (*F*(1,24) = 1.035, p = 0.319, ηp2 = 0.041).

## Discussion

The objective of this study was to investigate spatial mapping of touch in the deaf using the crossed-arm TOJ task. Results for the task revealed that deaf participants had a higher mean PCD group score, indicating more TOJ errors when crossing the arms, compared to hearing control group members. Results from the non-speeded reaction times for the crossed-arm task revealed no difference between groups. These results suggest that a period of deafness permanently alters spatial mapping of touch, but does not alter the time required to provide that judgment.

Many deaf participants provided inverted TOF responses when they crossed their arms (see Fig 1), which could be interpreted as a misunderstanding of the instructions. However, all participants provided verbal confirmation for understanding the task demands before and during the experiment, for both crossed and uncrossed postures. We also maintained a strict task comprehension inclusion criterion. Results from any participant demonstrating misunderstanding of task demand after the instruction sessions would have been removed from analysis. Thus eliminating the possibility of a misunderstanding, the increased error rate could be associated with the widely reported changes in body-related processes for deaf individuals (for review see ref [16]). More specifically, deaf individuals have been found to have significantly lower temporal tactile discrimination abilities [28]. It seems unlikely that the larger error rates stem from participants not understanding the task requirement due to our strict inclusion criteria.

Our results provide an important insight on deafness and the spatial mapping of touch for SOA up to 400 ms. However, future analysis on deaf participants will benefit from longer SOAs to reveal if deaf error rates are along an N-curve [3], [27], or an inverted psychometric function. It would also be worth investigating with methodologies where answers are given manually or vocally [29], as foot answers can lead to increased error rates [15].

Our findings are consistent with the crossed-arm TOJ results from Noel & Wallace [15] where participants could be temporarily deprived of either auditory or visual information. Their results suggest that only a period of short-term auditory deprivation lead to an increase in crossed-arm TOJ. This suggests that even short-term auditory deprivation is sufficient to alter spatial mapping of touch. Results from Noel & Wallace [15] also suggest that short-term visual deprivation can lead to a non-significant tendency towards reduced crossed-arm TOJ errors. Similarly, Röder et al. [8] found that congenitally blind participants had significantly less cross-arm TOJ errors than a sighted control group. It thus seems that vision, unlike audition, requires a longer period of sensory deprivation, perhaps even a congenital blindness, to lead to a significant different in crossed-arm TOJ.

Indeed, two studies have now indicated unimpaired crossed-arm performance in the early blind adults [8] [14]. This unimpaired performance of early blind adults has been suggested by Crollen et al. [14] to reflect that touch localization in this population relies mainly on the internal reference frame. The alignment of external and internal frames of references seen in sighted controls could hinge on early experience with seeing and feeling the arm interact with the environment. Our results with deaf individuals suggest an opposite alignment of frames of reference. Deafness thus seems to shift the balance for frames of reference leading to a heavier reliance on the external frame of reference for this touch localization task.

Yamamoto and Kitazawa [3] proposed that hands must be localized in space before the temporal order can be determined. In the crossed hands posture with short SOAs, the stimulations occur before remapping is completed, leading to the higher error rates. Our results suggest that this remapping takes longer in deaf individuals as their error rates in the crossed posture are higher than normal hearing-individuals. In contrast, Shore et al. [4] proposed that the internal and external frames of reference remain active after a frame of reference transformation. Per this hypothesis, the higher error rate found in the crossed-arm condition would be attributed to a greater cognitive effort required to resolve conflicting frame of reference information. As it takes time for information from the first stimulation to be localized, if the second stimulus is presented before the first is located, TOJ errors can occur. Were the Shore et al. hypothesis the only factor explaining the lower performance in the crossed-arm condition, we would expect both reaction times and error rates to be higher for deaf participants. However, an analysis of mean group unspeed reaction times failed to reveal a significant difference between deaf and hearing groups.

The effects of deafness on tactile abilities are highly variable. Some investigations on body perception in the deaf have reported no differences in tactile perception (see, e.g., refs [30] and [31]), while others have suggested improvements [32, 33] or even declines [28]. Investigations on abilities closer related to the cross-arm TOJ task on movement and posture have revealed a more consistent effect of deafness. These results suggest that deafness leads to impairments in tasks related to motor behavior or action (for review see ref [16]). Our results show that even in the absence of a motor component, deaf individuals can experience difficulties in tasks involving correctly judging the position of their body in space. This altered spatial mapping of touch provides a direction to better explain the deficits in tasks related to motor behavior or action in the deaf. The impact of individual characteristics of deafness on this reported altered spatial mapping of touch also merits further investigation.

These results are the first to investigate the interaction of internal and external frames of references in deaf individuals. The posterior parietal cortex (PPC) has been suggested to play a key role in the interaction of frames of reference in hearing individuals [34]. Several studies have demonstrated significant changes to PPC activation for visual stimuli in the deaf [35–37]. While these studies investigated visual processes, they highlight a plasticity in the PPC caused by a period of auditory deprivation. These neuroimaging studies suggest an increased presence of visual information in the PPC in the absence of auditory input. As vision represents information from an external frame of reference, this increased importance of visual information in the PPC could help explain why deaf participant provided externally-based responses for the TOJ task. Future studies are required to better understand the cortical mechanisms underlying this effect and the role of the PPC for the increased TOJ error rate in deaf individuals.

Participants all had similar onset of hearing loss, duration of hearing loss, hearing aids use, and modes of communication. These factors have been revealed to critically impact plasticity and performance in the deaf (see, e.g., ref [38]). Further studies should investigate the link between TOJ task performance and hearing gain using information including hearing thresholds with amplification, hearing aid data logging, and hearing aid adjustments parameters. We acknowledge that these factors have not been measured in our study and while this impact is not fully yet understood, it can impact plasticity leading to altered performances. Also, future research needs to examine the effect of these characteristics on the task results and also to evaluate whether there exists a critical period during which auditory input is required for normal-like spatial mapping of touch. Finally, in the present study, most deaf participants had vestibular impairments. Since vestibular function has an influence on body-related processes, such as body awareness and perception (for review see ref [39]), the deficit of the vestibular function might explain some of the results. Moreover, investigations using passive body rotation revealed an impact of rotation on TOJ [40]. The impact of vestibular impairment on the representation of the body in space needs to be explored further in order to disentangle if the lack of postural control observed in the congenitally deaf is the result of early auditory deprivation or vestibular impairment.

## Supporting information

S1 File Complete data set. Individual scores for each SOA and individual PCD scores for each hearing (n = 13) and deaf participants (n = 13).
(XLSX)
